# Stress Distribution within the Peri-Implant Bone for Different Implant Materials Obtained by Digital Image Correlation

**DOI:** 10.3390/ma17092161

**Published:** 2024-05-06

**Authors:** Ragai Edward Matta, Lara Berger, Moritz Loehlein, Linus Leven, Juergen Taxis, Manfred Wichmann, Constantin Motel

**Affiliations:** 1 Dental Clinic 2-Dental Prosthodontics, University Hospital Erlangen, Glueckstrasse 11, 91054 Erlangen, Germany; lara.berger@uk-erlangen.de (L.B.); msl98@gmx.de (M.L.); linus_leven@yahoo.de (L.L.); claudia.ehrhardt@uk-erlangen.de (M.W.); constantin.motel@uk-erlangen.de (C.M.); 2 Clinic for Oro- and Maxillofacial Surgery, University hospital Regensburg, Franz-Josef-Strauß-Allee 11, 93053 Regensburg, Germany; juergen.taxis@ukr.de

**Keywords:** dental implants, implant biomechanics, peri-implant stress distribution, implant materials, implant loading, optical measurement

## Abstract

Stress distribution and its magnitude during loading heavily influence the osseointegration of dental implants. Currently, no high-resolution, three-dimensional method of directly measuring these biomechanical processes in the peri-implant bone is available. The aim of this study was to measure the influence of different implant materials on stress distribution in the peri-implant bone. Using the three-dimensional ARAMIS camera system, surface strain in the peri-implant bone area was compared under simulated masticatory forces of 300 N in axial and non-axial directions for titanium implants and zirconia implants. The investigated titanium implants led to a more homogeneous stress distribution than the investigated zirconia implants. Non-axial forces led to greater surface strain on the peri-implant bone than axial forces. Thus, the implant material, implant system, and direction of force could have a significant influence on biomechanical processes and osseointegration within the peri-implant bone.

## 1. Introduction

The replacement of missing teeth with osseointegrated dental implants has been an established dental procedure since the late 1960s [1]. With the ever-increasing interest and innovations in the field of treatment planning and implementation, research in the field of biological–mechanical relationships is progressing [2]. The mechanical tension that acts at the junction of dental implants and peri-implant bone can lead to micromovements of the dental implants and is considered to influence osseointegration [3,4,5]. This aspect belongs to the broad field of dental biomechanics, the understanding and influence of which are of great importance for the long-term success of dental implants [6]. The transmission of force from implant to bone and, thus, the mechanical stress at the implant–bone junction depend on the direction of the applied force (axial vs. non-axial), the length and diameter of the implant, the design of the junction, and the quality of the bone [7,8,9,10]. Several different implant materials are currently being scientifically investigated or already in clinical use. The most common are titanium and zirconium oxide ceramic (zirconia) [11]. Titanium is the gold standard, as it is by far the best-studied implant material since its first use in the late 1960s [1,12,13]. Its advantages are excellent biocompatibility and well-studied and predictable osseointegration [14,15,16]. In addition, due to the very widespread use of dental titanium implants in dental practice, many different implant systems are available, which can be flexibly selected according to the patient’s situation [17]. The disadvantages include limited esthetics, particularly in the case of a high smile line with exposed implant shoulders, such as in the case of soft tissue loss or a very thin mucosa through which titanium implants can show [18]. Furthermore, the modulus of elasticity of titanium (~110 GPa) is significantly higher than that of cortical bone, which has a modulus of elasticity of ~13 GPa [19,20]. Over the last 15 years, zirconia has become established as an implant material [21]. This material also offers very good biocompatibility, and the absence of a microgap in one-piece zirconia implants is also seen as an advantage, in conjunction with reduced biofilm [22]. With the exception of very thin mucosal layers, Jung et al. found no color difference in the area of the gingival margin after implantation of zirconia implants in the porcine jaw [18]. In contrast, exposed portions of zirconia implants can lead to esthetically unsatisfactory results due to the appearance of an unnatural coloration in the event of mucosal recession [23]. Another disadvantage for a long time was that zirconia dental implants were mostly manufactured in one piece (i.e., the implant–abutment complex consisted of a single workpiece). This means that the possibility to individualize implant therapy was low. In a regular case, it was only possible to choose from differently configured, prefabricated implant–abutment complexes [24,25]. Two-piece implant systems made of zirconia are now also available [26]. Another disadvantage of zirconia implants is their extremely high modulus of elasticity (~210 GPa) relative to the modulus of elasticity of bone [27]. Therefore, this implant material could lead to high stress peaks in the peri-implant bone [28]. In addition, zirconia implants were established in the dental field only a few years ago and are not as well studied as titanium implants. Little is currently known about the influence of different implant materials on the stress distribution in the peri-implant area under masticatory loading. Studies simulated the stress distribution in this area using the finite element method in a three-dimensional computer model, but this procedure is subjected to the limitation of the mathematical simplification of virtual test models [29]. The direct, three-dimensional measurement of stress distribution on the peri-implant bone under simulated masticatory force application was established for the first time by this working group. Surface changes during measurement by the ARAMIS system from Carl Zeiss GOM Metrology GmbH (Braunschweig, Germany) under masticatory loading correspond to the accuracy of strain gauges [30]. The aim of the present study was to investigate the influence of different implant materials, specifically, titanium and zirconia, on stress distribution in the porcine jaw. For this purpose, zirconia implants and titanium implants were loaded axially with masticatory forces of 300 N at an angle of 30° and the deformation of the bone surface was detected using the ARAMIS system. As null hypotheses, we assumed that the implant material and the masticatory force direction had no influence on stress distribution in the peri-implant bone under masticatory loading.

## 2. Materials and Methods

**Figure 1:**
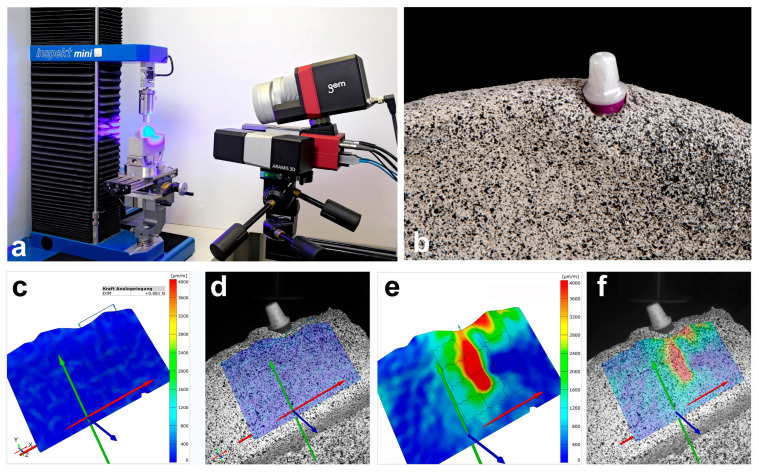
Overview of the experimental setup and presentation of the computer-aided evaluation in the study presented. (**a**) Experimental setup with the prepared bone, inserted implant, universal power machine on the left and ARAMIS camera system on the right (consisting of two cameras and a blue light lamp); (**b**) macrograph of the bone preparation with an inserted zirconia implant and a stochastic contrast pattern; (**c**,**d**) exemplary representation of the ARAMIS Professional software without force application (test setup with force direction of 30°); (**e**,**f**) exemplary representation of the ARAMIS Professional software with force application of 300 N (test setup with force direction of 30°, changes in shape in green, yellow, and red).

Five pieces of bone were prepared from the dorsal ramus region of five pig mandibles and embedded in plaster (Fujirock^®^, super hard stone type 4, GC Europe N.V., Leuwen, Belgium). It was necessary to keep the sample size as small as possible, as the porcine mandibles available for scientific use were limited. The relatively small sample size of 5 specimens allowed for a meaningful statistical evaluation in this context. Due to the nature of porcine bone, there were individual differences in the shape of the bone pieces, which were approximately 8 cm wide and 6 cm high. The drill studs for the two implants examined in each piece of bone (titanium: bone-level implant, diameter 4.1 mm, length 10 mm; zirconia: ceramic implant monotype, diameter 4.1 mm RD, length 10 mm; Straumann GmbH, Freiburg, Germany) were prepared according to the supplier’s instructions. Regarding the bone dimension around the implant after insertion, it varied individually. Typically, approximately 1 mm of bone remained buccally and orally around the implant site. These variations reflect the natural variability in the bone structure. The bone surface was then sprayed with an acrylic resin-based varnish (Sparvar color spray, Spray-Color GmbH, Merzenich, Germany) and a graphite varnish (CRC Industries Deutschland GmbH, Iffezheim, Germany) to create a stochastic contrast pattern. For both implant types examined, five implants were inserted individually into one bone block each and loaded by a compression testing machine (inspekt mini, Hegewald & Peschke Mess- und Prüftechnik GmbH, Nossen, Germany) with a 300 N force in two different directions (0°, “axial” and 30°, “non-axial”). The sample size of five implants per implant type was chosen deliberately. This number allowed statistical comparisons to be made with confidence, as it met the requirement for meaningful statistical analysis. For the titanium implants, insertion posts were inserted and screwed into place to support the masticatory load. The zirconia implants were a one-piece implant system. In the 0° test series, the force was applied in the direction of the longitudinal axis of the implant. In the 30° test series, the force was applied with an inclination of 30° with respect to the longitudinal axis of the implant (Figure 1a,b). The strain on the surface of the bone caused by the load on the implants was measured using the ARAMIS 3D optical camera system (Carl Zeiss GOM Metrology GmbH, Braunschweig, Germany), a non-contact optical three-dimensional deformation measurement system that is able to analyze movements and deformations through digital image correlation [31]. The displacement was calculated by assigning gray value distributions in the deformed image to gray value distributions in the undeformed reference image. The ARAMIS system was positioned orthogonally to the course of the examined bone. The calibration and distance between the peri-implant measurement area and the lenses of the ARAMIS system corresponded to the manufacturer’s specifications. The ARAMIS Professional software Version 2020 (Carl Zeiss GOM Metrology GmbH, Braunschweig, Germany) was used to examine the technical strain in the X and Y directions, as well as the main deformation. In the study, the *X*-axis corresponded to the mesio-distal orientation, whereby the left side in the test arrangement was defined as mesial. Accordingly, the force was applied in the 30° test series from a distal direction. The *Y*-axis corresponded to the corono-apical dimension. Figure 1c,d show the visual surface representation using the ARAMIS system without force application and, therefore, without deformation (plain blue region of interest in Figure 1d). An exemplary visual evaluation with force application of 300 N is presented in Figure 1e,f.

The numerical evaluation was carried out in the form of absolute measured values using raw data tables from the ARAMIS Professional software, which were transferred to Excel (Microsoft Corporation, Redmond, WA, USA). To differentiate the strain distribution, the peri-implant bone was divided into a total of 12 equally sized areas, which were arranged in four three-part rows. The dimensions resulted from the macro design of the implant. The width of each area was the same as the individual implant diameter, while the length of the area was one-third of the implant’s individual length. One row of measurement areas was added at the implant’s apex. The described arrangement led to four measurement areas on the left (a,d,g,j), right (c,f,i,l), and in a projection (b,e,h,k) of the implants examined. The measurement areas b, e, and h reflect the projection of the implant on the surface of the bone. The areas j,k,l represent the area at the implant´s apex. To enable statistical comparisons between the implant types, the deflection values were averaged within the specified measurement areas. The mean values are presented descriptively for the two implant types, separated by force and loading direction, and were compared non-parametrically between the implant types using single-factor analysis of variance (ANOVA) for statistical analysis. In addition, the percentage distribution of the fields with the highest deflection in terms of an averaged normalized relative deflection per field is presented graphically for both implant types and both force directions in relation to each individual implant position.

## 3. Results

**Figure 2:**
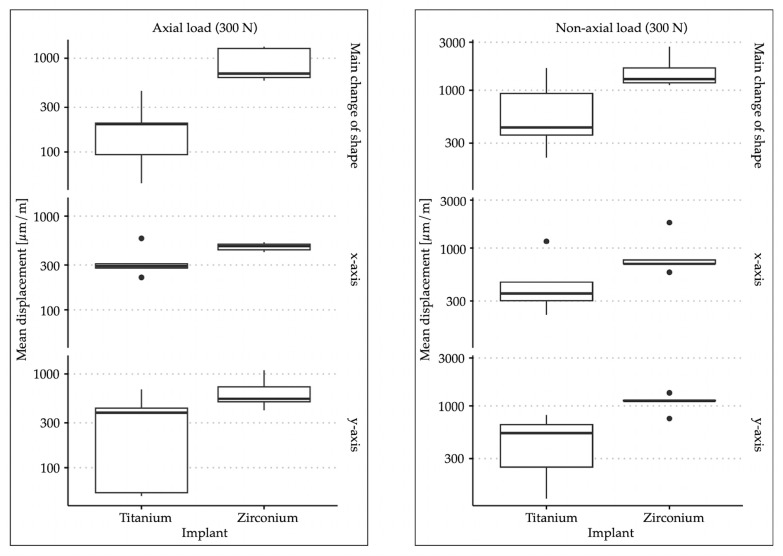
Box–whisker plots of the surface shape changes in the three investigated dimensions with axial and non-axial force application for both implant materials.

The comparison of surface deformation of the peri-implant bone under axial loading showed a significantly greater main deformation when a force of 300 N was applied on the zirconia implant; the same applied to the deformation in the X and Y directions (more precisely). The titanium implant showed an average main deformation of 198.38 µm/m, a deformation of 336.02 µm/m in the *X*-axis, and a deformation of 320.90 µm/m in the *Y*-axis. The main deformation of the peri-implant bone with the zirconia implant amounted to 898.95 µm/m. The deformation in the *X*-axis was 471.83 µm/m, and that in the *Y*-axis 654.69 µm/m. The overall descriptive statistics are shown in Table 1. Figure 2 shows the data in the form of a box–whisker plot. When loading was applied at an angle of 30° to the longitudinal axis of the implant, a significantly greater change in the main shape was observed in the area of the peri-implant bone with the zirconia implant. There were also significantly greater changes in shape in the X and Y directions. In this case, the titanium implant was associated with in a main deformation of 720.77 µm/m, with a deformation of 501.09 µm/m in the *X*-axis and 475.05 µm/m in the *Y*-axis. For the zirconia implant, the main deformation was 1601.46 µm/m, with a deformation of 906.31 µm/m in the *X*-axis and 1095.38 µm/m in the *Y*-axis. The descriptive statistics regarding the force application at an angle of 30° can also be found in Table 1, and the graphical representation in Figure 2. Table 2 provides the *p*-values from the non-parametric analyses of variance.

**Figure 3:**
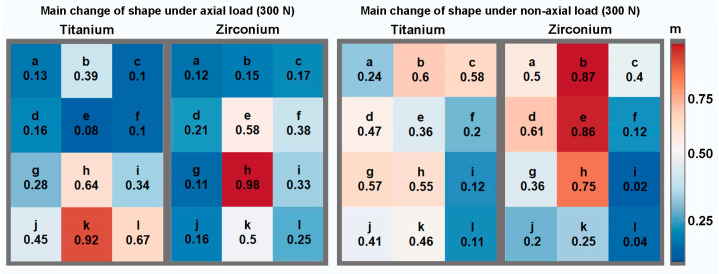
Relative representation of the main change in shape according to the measurement areas with axial and non-axial loading for both implant materials.

**Figure 4:**
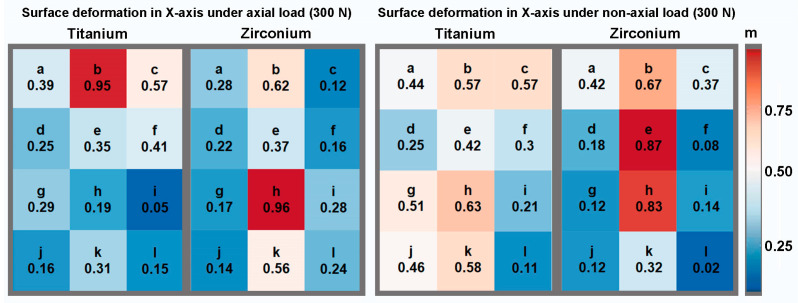
Relative representation of the surface deformation in the X-axis according to the measurement areas under axial and non-axial loading for both implant materials.

**Figure 5:**
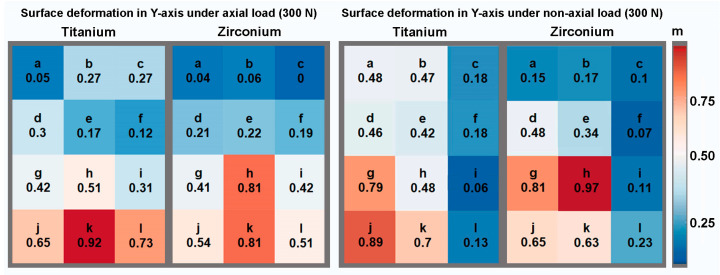
Relative representation of the surface deformation in the Y-axis according to the measurement areas under axial and non-axial loading for both implant materials.

To illustrate the results, Figure 3, Figure 4 and Figure 5 show the percentage shape change in relation to the peri-implant measurement areas for the axial and non-axial examinations, respectively. This is the percentage of the average change in shape over the individual test series in relation to the largest change in shape within the test series. For example, a change in shape of 0.9 means that an average change in shape of 90% was calculated in the specified measurement area, measured against the highest individually measured change in shape within the test series. The largest deformation of the main shape was found in the axial direction for both implant materials; in the case of the titanium implant, this was concentrated apically to the implant (measurement area k), while for the zirconia implant, it was concentrated in the area of the apical third in relation to the implant axis (measurement area h). For the change in shape in the X and Y directions, relatively symmetrical results were obtained for the axial load in relation to the implant axis. On the X-axis (mesio-distal direction), the greatest changes in shape were calculated in the cervical third for the titanium implant and in the apical third for the zirconia implant, in the projection of the implant in each case. On the Y-axis (apical–cervical direction), the largest changes in shape were calculated further apically for both implant materials. Even in the test series with a force application at an angle of 30°, the largest deformation changes, including those on the X- and Y-axes, were found in the projection of the longitudinal axis of the implant. Overall, when loading at an angle of 30°, an asymmetrical distribution of the size of the deformation changes was observed in the individual observations. Larger changes in shape could be calculated on the mesial side of the peri-implant bone, opposite to the load.

## 4. Discussion

The 3D optical image correlation used in the present study to detect changes in the shape of superficial bone due to stress induction is already well established in dentistry [32,33,34]. The transfer of this technology to the measurement of peri-implant bone during masticatory force application was presented as part of a pilot study in 2021 [30]. Good repeatability and measurement stability can be attributed to the method. The present study is the first to use this technology to investigate peri-implant stress under masticatory force application as a function of the implant material. Very few studies investigated stress propagation in bone using the finite element method [11]. A finite element method-based study showed that implant materials with a lower modulus of elasticity cause greater stress within the cortical bone under a chewing force application of 100 N compared to those with a higher modulus of elasticity. A chewing force simulation on one-piece implants made of titanium, zirconia, and various PEEK materials was investigated. In contrast, a study by Haseeb et al. comparing carbon-fiber-reinforced PEEK implants with conventional pure-titanium implants showed a comparable stress distribution in the peri-implant bone [11,35]. When considering these two studies, a particular limitation of the finite element method becomes clear. It is a simulative research method that is dependent on the parameter definitions and therefore makes it fundamentally difficult to compare different studies [36]. In this context, the data collected in the present study showed contradictory results. With regard to the main shape change, significantly greater superficial changes in the shape of the peri-implant bone were found with the zirconia implant. Notably, the measurement method used in this study, as a direct, optical procedure, differs fundamentally from the simulative method of the finite element method. In addition, different masticatory forces were considered. At this point, it should be noted that the present data were collected using an avital bone preparation. This means that osseointegration could not take place, and the results, therefore, represent a situation of primary stability or immediate loading. The results related to the axial force application revealed an overall symmetrical distribution around the examined implants. The different localization of the largest deformation changes depending on the implant material was striking. In the case of the zirconia implant, the largest changes in shape were found in the apical third of the projection of the implant, in relation to both the main change in shape and the X- and Y-axes. In contrast, in the case of the titanium implant, a more heterogeneous distribution of the largest shape changes was observed in the three dimensions. This indicated an overall greater local concentration of stress in the area of the peri-implant bone with the zirconia implant than with the titanium implant. Conversely, the results indicated a more even distribution of stress in the peri-implant bone in the case of the titanium implant. This was reflected in the comparatively smaller changes in shape in the case of the titanium implant and could have a positive effect on osseointegration. The stronger concentration of stress in implants with a comparatively higher modulus of elasticity (“stress isolation”) was recently demonstrated by Masoomi et al. in a finite element analysis [37]. In this context, this shows a very good comparability of finite element analysis and digital image correlation. In particular, further investigation of the geometry of the implants and their effect on stress distribution in the surrounding bone will be clinically relevant in the future. The results of the analysis of force application at an angle of 30° suggested that the strain in the dimensions examined was closer to the projection of the implant in the peri-implant bone with the zirconia implant than with the titanium implant. For both implant materials, greater elongation was observed in all dimensions on the side contralateral to the force application compared to the ipsilateral side. In contrast, more eccentric strain distributions were observed for the titanium implant. These were comparable to the those obtained with axial force application, more homogeneous over the measurement area of the peri-implant bone, and less pronounced overall. In principle, the greater modulus of elasticity of the zirconia implants could lead to a more direct transfer of masticatory forces into the peri-implant bone, which may be reflected in a greater surface deformation. However, despite the significant differences in deformation, the overall differences were small. Incidentally, the zirconia implant was a one-piece implant system. In contrast, the titanium implant consisted of a screw-retained implant–abutment complex, which represents a combination of implant and abutment. This im-plant–abutment connection could potentially lead to reduced stress distribution into the peri-implant bone. This could have come into play, particularly with a masticatory force application at a 30° angle. A greater difference in the change in shape between the titanium and the zirconia implants was found for each of the three investigated changes in shape when force was applied at a 30° angle than when an axial chewing force was applied. To the best of our knowledge, no directly comparative data are available regarding stress distribution in the peri-implant bone with single-piece implants and implant–abutment systems. In this context, Tribst et al. were able to demonstrate that a semi-rigid implant–abutment connection can lead to a lower stress propagation in the peri-implant bone compared to a rigid connection, which fundamentally supports the results of the present study [38]. The overall greater and asymmetrical stress transmission into the peri-implant bone with non-axial masticatory force transmission could also argue against the immediate or early loading of implants in the anterior region, as it was already shown that non-axial forces can lead to greater deflections even with relatively low loading [39]. Such micromovements can lead to the formation of fibrous tissue between the implant and the bone in implants that are not yet fully osseointegrated and thus to implant loss [40,41,42].

For the clinical application of dental implants, it is imperative to meticulously examine the strategies by which peri-implant stress can be mitigated during the period prior to osseointegration. This phase is characterized solely by the attainment of primary stability, which critically impacts the subsequent healing processes. Peri-implant bone’s stress distribution plays a pivotal role in the bone’s healing trajectory. When the loading exceeds the mechanical tolerance of the peri-implant bone, pathological outcomes such as cartilagogenesis or fibrous tissue formation may ensue, highlighting the detrimental effects of excessive mechanical loading during the initial healing phase [43,44]. This study posits that a uniform stress distribution within the peri-implant bone may confer therapeutic benefits. Consequently, it is evident that comprehensive investigations into the specific patterns of peri-implant stress distribution and its influence on bone integrity are essential. These studies are crucial for formulating precise clinical guidelines concerning the immediate loading of dental implants, thereby optimizing treatment outcomes.

The design of the present study is subject to various limitations. First, an in vitro procedure was used that cannot fully reflect the actual conditions in the oral cavity. In addition, the standardization of chewing force initiation, which was necessary for experimental reasons, limits the possibility of generalizing the results of the present study. Furthermore, the methodology used only detects superficial changes in shape and does not allow for any direct transfer to processes within the bone. Overall, the results presented should be supplemented by further investigations.

## 5. Conclusions

In the present study, the implant material and implant system significantly influenced the deformation of the superficial peri-implant bone under masticatory force application. Zirconia caused locally more concentrated stress propagation into the peri-implant bone, with relatively small overall strain. In addition, non-axial forces led to greater peri-implant stress than axial masticatory forces, and the strain was greater on the contralateral side of the force direction than on the ipsilateral side. In connection with physiological bone remodeling, which loads on dental implants may have positive or negative influences on the physiological processes of bone metabolism remains to be investigated.

## Figures and Tables

**Table 1:** Descriptive statistics of the mean surface shape changes in the investigated dimensions regarding both implant materials ^1^.

Material	Angulation	Main Change in Shape		Change in Shape in *X*-axis		Change in Shape in *Y*-axis	
		Mean	SD	Mean	SD	Mean	SD
Titanium	0°	198.38	155.57	336.02	139.62	320.9	270.41
	30°	720.77	594.95	501.09	383.49	475.05	287.1
Zirconium	0°	898.95	373.53	471.83	47.25	654.69	271.02
	30°	1601.46	661.08	906.31	499.68	1095.38	216.24

^1^ All measured values are in µm/m.

**Table 2:** *p*-values regarding the differences between the two implant materials in relation to the mean surface shape changes in the three dimensions investigated (calculation by ANOVA).

Angulation	Dimension	*p*-Value
0°	main change in shape	0.009
	*X*-axis	0.1172
	*Y*-axis	0.0758
30°	main change in shape	0.0472
	*X*-axis	0.0758
	*Y*-axis	0.0163

## Data Availability

The underlying data can be requested via the correspondence address.
